# A Saudi Arabian Public Health Perspective of Tuberculosis

**DOI:** 10.3390/ijerph181910042

**Published:** 2021-09-24

**Authors:** Abdullah A. Saati, Muhammad Khurram, Hani Faidah, Abdul Haseeb, Marcello Iriti

**Affiliations:** 1 Department of Community Medicine & Pilgrims Healthcare, Faculty of Medicine, Umm Al-Qura University, Makkah 24382, Saudi Arabia; aaasaati@uqu.edu.sa; 2 Department of Pharmaceutics, Faculty of Pharmaceutical Sciences, Abasyn University, Peshawar 25000, Pakistan; 3 Department of Microbiology, Faculty of Medicine, Umm Al Qura University, Makkah 24382, Saudi Arabia; hsfaidah@uqu.edu.sa; 4 Department of Clinical Pharmacy, College of Pharmacy, Umm Al Qura University, Makkah 24382, Saudi Arabia; amhaseeb@uqu.edu.sa; 5 Department of Agricultural and Environmental Sciences, Università degli Studi di Milano, 20133 Milano, Italy; 6 Phytochem Lab, Department of Agricultural and Environmental Sciences, Università degli Studi di Milano, 20133 Milano, Italy; 7 Center for Studies on Bioispired Agro-Environmental Technology (BAT Center), Università degli Studi di Napoli “Federico II”, 80055 Portici, Italy; 8 National Interuniversity Consortium of Materials Science and Technology (INSTM), 50121 Firenze, Italy

**Keywords:** *Mycobacterium tuberculosis*, hajj, umrah, pilgrims, MDR, XDR, TDR

## Abstract

Tuberculosis is a global health challenge due to its spreading potential. The Kingdom of Saudi Arabia (KSA) faces a challenge in the spread of tuberculosis from migrant workers, but the foremost threat is the huge number of pilgrims who travel to visit sacred sites of the Islamic world located in the holy cities of Makkah and Al Madina. Pilgrims visit throughout the year but especially in the months of Ramadan and Zul-Hijah. The rise of resistance in *Mycobacterium tuberculosis* is an established global phenomenon that makes such large congregations likely hotspots in the dissemination and spread of disease at a global level. Although very stringent and effective measures exist, the threat remains due to the ever-changing dynamics of this highly pathogenic disease. This overview primarily highlights the current public health challenges posed by this disease to the Saudi health system, which needs to be highlighted not only to the concerned authorities of KSA, but also to the concerned global quarters since the pilgrims and migrants come from all parts of the world with a majority coming from high tuberculosis-burdened countries.

## 1. Introduction

World tuberculosis (TB) day, on March 24 each year, commemorates the day when Dr Robert Koch announced his historic discovery of *Mycobacterium tuberculosis* (MTB) as the cause of TB [1]. However, every 20 s, somewhere in the world a TB patient dies [2]. The rates of morbidity and mortality steadily fell in the developed countries during the 20th century due to better treatment and prevention measures along with good public health practices. Unfortunately, this descent stopped and started increasing again in the mid-1980s.

Following inhalation as respiratory droplets, and on reaching the distal alveoli, the MTB get ingested by the alveolar macrophages as well as other phagocytic cells, where they interfere in the normal maturation of the phagosome and reside in an early endosome-like compartment. The disease progression and resolution can be a four-staged process. In the first 3–8 weeks, when the MTB implants itself, it disseminates to regional lymph nodes resulting in the Ghon complex. The following stage usually lasts for 3 months in which hematogenous circulation carries bacteria to other organs as well as to other parts of lungs. This dissemination may result in miliary TB or cause meningitis, both of which have acute and occasionally terminal consequences. In the third stage the pleural surfaces may become inflamed (condition termed pleurisy), lasting 3–7 months (this stage may not appear for as long as 2 years). Bacteria and their components may interact with T-lymphocytes (CD4^+^) that release inflammatory cytokines following their proliferation. In this scenario the dendritic cells also play an active role since they are better antigen presenters (in this case MTB antigens) than the macrophages, and due to their migratory potential, thus playing an active role in dissemination. This is followed by the resolution phase in which the disease does not further excel but is marked through the formation of extrapulmonary lesions in, e.g., joints and bones. It is pertinent to mention that immunocompromised patients, especially those infected with HIV, have a 50% propensity of TB acquisition (as new cases) and have same chances of reactivation as well, which in most cases quickly goes on to the active disease phase with greater lung involvement resulting in extensive damage marked by necrosis and bleeding. As far as the pathogenesis is concerned, MTB has severe consequence at the usual site of infection, i.e., lungs, causing extensive damage that may result in fatal consequences due to anoxic state, which ensues due to damage to the parenchymal cells in the lungs. These cells play a central role in oxygen uptake. Moreover, obstruction in the bronchioles due to granulomatous growth as well as the rupture of the liquified granulomas, result in blood release that is coughed out or expectorated as vomitus. The organism may reach the meningeal membranes of the brain, resulting in death due to inflammation of brain tissues. In this context MTB has a central role in the induction of inflammatory responses in the host, which contribute to infection control but also inflict wide tissue damage. Virulence factors like cathepsin D [3] have been correlated to granulomas’ liquefaction, but other factors, like apoptosis of the macrophages [4], may contribute to tissue injury. Moreover, the tissue necrosis factor-α (TNF-α) has been related to TB progression along with other cytokines. There are complex immune responses as far as the virulence of MTB and the cytokines levels are concerned. An optimal balance is required for the immunomodulators to cope with the challenge of MTB, which has wide genetic diversity and clinical implications.

KSA is one of the most pivotal Muslim countries due to the presence of two holy sites of worships in the Makkah and Al Madina regions. Muslims from around the world visit the sacred places year-round, with peaks during the annual Hajj pilgrimage in the month of Zul Hijah (according to lunar calendar). Moreover, for Umrah, a shorter pilgrimage undertaken throughout the year, pilgrims number swells especially in the month of Ramadan (as per lunar calendar). This situation has every propensity to support the spread of infectious diseases, especially respiratory tract infections. The recent spread of COVID-19 is an example of how respiratory tract-affecting pathogens may spread and cause enormous damage. Although MTB does not spread as rapidly, it still has the potential of becoming a globally spreadable organism, which, due to its ever-increasing resistance issues, is becoming a serious public health threat. This review encompasses different aspects associated with this organism that are pertinent not only for the health authorities of KSA but all those concerned from countries where pilgrims and migrants arrive. It highlights the epidemiological features of not only the KSA but also neighboring countries with respect to MTB. The disease, brief genetic aspects, drug resistance issues, treatment options, and public health concerns are discussed highlighting the problems and the possible remediations from the perspective of KSA.

In a recent Ethiopian study, mortality predictors included people of older age, having low body weight especially at the start of antitubercular therapy (ATT), living in rural areas, retreatment of the patient, having extrapulmonary tubercular infection, and having human immunodeficiency virus (HIV) co-infection [5]. Similarly, in another Ethiopian study, extrapulmonary tuberculosis (EPTB) co-infection was a common finding in HIV seropositive patients. Moreover, the presence peaked in individuals with high levels of immune suppression. EPTB is a major opportunistic challenge in HIV positive patients with most frequent involvement of lymph nodes followed by pleural and dissemination forms. The majority of EPTB reports were recorded in the first years of follow-up with lower baseline helper T-lymphocytic cells (CD4^+^; *CD* = *Cluster of differentiation*) counts and anemia found to be independent risk factors for EPTB. Those patients who were taking antiretroviral therapy as well as isoniazid (INH) were shown to have reduced risk of EPTB [6].

In this context, TB monitoring is crucial throughout the course of patient treatment, starting from diagnosis. In another Ethiopian study, higher prevalence of TB with Rifampicin (RIF) resistance and TB/HIV coinfection has been reported. Emphasis was given on the collaborative and intensified prevention of TB and HIV. Furthermore, need of extensive monitoring of HIV was suggested to minimize co-infections and associated problems [7]. In another study, HIV seronegative TB infection showed marked CD4^+^ lymphocytopenia, neutrophilia, and monocytosis. Females were found to be more prone to these effects with conditions being more prominent in advancing age groups [8].

The HIV and TB co-infection is a significant cause of mortality in HIV seropositive patients and is becoming a new public health challenge especially for TB control programs. The need of initiating HIV and TB collaborative programs at a global level has been emphasized [9]. Social determinants play a vital role in the mortality of the coinfection of TB and HIV. As well as medical interventions, an improvement in social determinants is required [10]. Moreover, children have a tendency to get affected by TB in cases of close contact, which highlights the significance of contact tracing. The prevalence of childhood TB with resistant strains is indicative of household contact [11], thus making screening of children essential in limiting the pediatric spread of TB. Moreover, for children, other sampling techniques need to be standardized as young children may not be able to expectorate [12].

Other mycobacterial challenges also need to be kept in mind. For instance, in a Serbian study, *M. chlonae* was detected in bronchoscopes used in pediatric patients. *M. fortuitum* was the causative agent for endocarditis in three heart-operated Serb children, attributed to the bovine pericardial patches that were used for ventricular septal defects [13].

Ocular TB presents a clinical challenge. The interferon-γ release assays that are used for its diagnosis primarily lack the specificity to differentiate between active and latent TB. Molecular diagnostics have been the key towards diagnosis as well as therapy initiation [14]. In this regard, the uveal tract in the eye, owing to its high blood supply, may serve as a common TB site without clinical evidence of systemic TB. Ocular TB may appear as a primary disease affecting the conjunctiva, cornea, and sclera that may progress secondarily to ocular tissues. Due to the presence of MTB antigens, disease may appear as a hypersensitivity reaction. With no confirmatory test for this disease, diagnosis is primarily based on clinical signs and symptoms alongside different investigations. To avoid blinding complications, a panel consisting of an ophthalmologist, pulmonologist, pathologist, microbiologist, and an internist is key to management of patient.

The risk of spread of multidrug resistant tuberculosis (MDR-TB) is also significant. Moreover, the need of newer diagnostic assays with high sensitivity is needed in order to evaluate the negative cases. There is a global resurgence of TB and MDR-TB, which is not confined to developing countries [15]. The use of antitubercular treatment in patients with TB uveitis finds low treatment failure compared to those patients in which there is choroidal involvement and viterous haze phenomena [16]. *M. tuberculosis* has the potential to directly infect the eye through antigenic mimicry that yields intraocular inflammation. Ocular involvement in TB patients is estimated to be in range between 1–4%, but may be as high as 10–26% in highly endemic regions like KSA. Nucleic acid amplification tests have been regarded as a powerful tool in the rapid detection of TB and have the additional advantage of detecting dormant mycobacteria in normal tissues [17].

Diabetes mellitus (DM) is associated with increased mortality during TB treatment especially in the second month of therapy. Therefore, routine screening of TB patients with DM is required [18]. There is high prevalence of HIV, TB, HIV, and TB in DM patients [19]. Although estimated patient mortality rate was lower than earlier reports, advanced age and associated comorbidities like renal failure, DM, chronic lung disease, HBV infection, and chronic heart failure (CHF) have significant correlation with hospitalized patients’ mortality rate with strong predictors included CHF, lung disease, and >65 years of age. The mortality rates can be lowered through rational therapy and management of disease and comorbidities, as well as adoption of better prevention policies [20]. Diabetic pulmonary TB patients show unfavorable treatment outcomes. It is imperative to associate TB and diabetic diagnostics and therapeutics. Early screening and consequent management for TB with diabetes should be started as early as possible, which will improve TB treatment outcomes [21]. The high prevalence of DM in the MDR-TB is a serious concern, and it presents as an independent risk factor for MDR-TB especially in primary cases. Patients having DM-TB comorbidity need to be diagnosed early and require intensive treatment, follow-up, and monitoring [22]. DM is a recognized comorbidity that accelerates TB and complicates its treatment. Smoking increases the risk of TB as well as mortality. Strict glycemic control, switching to an extremely drug resistant tuberculosis (XDR-TB) regimen from MDR-TB in preXDR-TB, and drug rehabilitation should be carried out in drug resistant TB patients [23]. Overall risk of TB in persons with DM is two to three times higher than the general population since chronic hyperglycemia has an association with treatment outcome and prognosis of TB. The increased risk of acquiring MDR-TB in DM patients needs further research [24]. Overall median global prevalence of DM in TB patients has been observed to be 16%. Alternatively, the TB median global prevalence in DM stood at 4.1%, thus indicating a high burden of DM in TB patients. The key variables included gender, advanced age, smoking, urban residence, poor glycemic control, sedentary lifestyle, and family history of DM and TB [25]. The sugar metabolism disorder increases the tissue sugar level, which encourages microbial proliferation. Resistance to MTB plummets as a consequence of DM. Nearly a third of the world population is latently infected with MTB and in developing countries 2–3 million lives each year are claimed by this disease. Diabetes may result in therapy failure, death during therapy, and disease relapse. Thus the TB control programs need to focus more on the treatment and monitoring of patients suffering from DM [26]. A recent Brazilian study found men to be more affected from TB and DM. Moreover, adolescents and older women were also shown to be at higher risk of having TB and DM [27].

The common presentation in the case of head and neck involvement of TB is cervical lymphadenitis, which is present in 70–90% cases, but TB has the propensity to cause effects on salivary glands, ear, oral cavity, larynx, and cervical spinal region. There is also the possibility of abscesses of the deep neck. Based on drug susceptibility assays, multidrug antitubercular regimens should be opted for treatment [28]. Tuberculosis may also result in meningitis that is difficult to differentiate from that of other bacterial origins. The treatment gets prolonged in cases of suspected or confirmed MDR-TB with further aggressive therapy under experienced specialized medical staff [29]. There is a global surge of spinal TB that is attributable to inadequate duration of treatment, improper dosing, and selection of chemotherapeutic agents. Furthermore, migration at the global level has been associated with spinal TB. This medical condition needs to be diagnosed and treated early, in order to prevent the complications, and also make the mobilization early so as to bring patients back to functional status [30]. Spinal TB may result in paradiscal involvement, destruction of the endplate, and masses of soft tissues. Magnetic resonance imaging (MRI) is an important tool in this respect that may help in the early diagnosis of the disease. It may also help in the evaluation of the success of chemotherapy as well as in the decision to carry out surgery. MRI examination can also be of significant value in the determination of healing that is indicated by a decrease in inflammatory soft tissue masses and a drop in the vertebral body marrow edema. Short-term therapy of 6-months duration may not be effective in curing spinal tuberculosis thus it will require an extensive therapy [31].

MDR-TB has also been reported to affect the women of childbearing age. This is of particular concern when the patients are pregnant and require administration of anti-TB therapy that not only affects the mother, but may also have serious consequences on the fetus [32]. Genital tuberculosis has been reported for significant morbidity with varying incidence rates. It may lead to infertility in females as a consequence of damaged to the fallopian tubes and endometrium [33].

End stage renal disease patients are at a very high risk of TB acquisition. Due to the lack of diagnostic techniques, peritoneal tuberculosis is an underdiagnosed ailment. It needs an early diagnosis and a prompt therapy of not less than 12 months in order to achieve better treatment outcomes [34].

All these ailments in association with TB present with not only significant health and clinical challenges, but also represent a daunting task for public health authorities of developed as well as developing and poor countries to build counter strategies. Some other forms of EPTB [35], are summarized in Table 1, highlighting the kind of TB challenges we are facing currently.

The etiological agent of TB is well known and characterized, but still this disease is having a global prevalence, incidence, and surging mortality worldwide. The mortality predictors include older age, low body weight, delayed start of ATT, rural living, retreatment patients, EPTB infection, low immune status, comorbidities, and coinfections especially for the HIV cases. In this context the HIV coinfection presents as a significant public health challenge particularly in the wake of M/XDR-TB thus requiring a persistent and extensive monitoring. Moreover, childhood TB is also a very concerning issue. Similarly, DM presents another huge challenge as it has an established role in increasing the mortality in TB patients thus necessitating its mandatory screening. Moreover, females of childbearing age have also been found to be at more risk compared to non-childbearing females. The problem gets graver with reports suggesting females to be more prone to TB compared to males. DM patients often present with other comorbidities, such as heart and renal diseases, thus making the treatment further complex with unfavorable outcomes. When it is seen in the context of M/XDR-TB the therapeutics it becomes more difficult to achieve the desired outcomes.

## 2. Epidemiological Features

TB eradication by 2030 is part of the Sustainable Development Goals (SDGs) by the World Health Organization (WHO). The top six TB burdened countries (India, Indonesia, China, Nigeria, Pakistan, and South Africa) of the world account for 60% of new cases. TB was one of the top 10 causes of death worldwide in 2015. The ambitious target set by the WHO can only be transpired into reality by strong governmental commitment and appropriate investments in research. Furthermore, the spread of drug resistant TB should be kept in picture as it poses a serious challenge to TB control programs [37].

India and China account for half of all the global TB cases [38] and their huge migrant force may influence the dynamics of the spread of tuberculosis. Similarly, in a current epidemiological report that encompassed 11 years of data from a northern Iranian province, pulmonary TB was observed to be the most prevalent form of TB [39]. In an Ethiopian six-year study, a declining trend in TB was observed, but cases represented the productive age group that influences not only the individuals but the country at large. HIV seropositive and MDR-TB were significantly associated with TB [40]. The western part of India has high prevalence of MDR-TB. The dominant mutations include codon 315 in katG and in rpoB codon 531. The previously treated cases showed more mutation diversity along with a higher number of unknown mutations [41].

Qatar is a country with a high rate of migration, with expatriates more likely to be infected from drug-resistant tuberculosis (DR-TB). In a recent Qatari study, pulmonary TB was found to be the predominant form while pleural TB remained the second major site. The MDR-TB was associated more with pulmonary TB. Complete success of MDR-TB treatment was attributed to strict adherence to Directly Observed Therapy (DOTs) for the entire time frame of treatment. However, the HIV and MDR-TB relation was not established, although the WHO recommends routine HIV testing as part of TB/HIV interventions offered to all TB patients [42]. The global TB report of 2016 gives a diverse burden with Kuwait leading the list with 200 million while UAE had 6.8 million and was at the bottom of the list. KSA, with 89 million, was considered a low-incidence country. This status did not get changed for three years. It is assumed that the countries of Gulf Health Council (GHC) will reach the pre-elimination by 2043. Although the GHC members have a better financed health system, including TB diagnosis, control, and treatment as its main components, challenges remain. Most importantly, GHC member countries have the highest comparative diabetes prevalence in the world with five members ranked among the top 10 by the International Diabetic Federation. Since diabetes has been found to increase a person’s TB acquisition risk, it is pertinent to develop a collaborative platform for controlling TB in diabetics. Similarly, the HIV patients’ risk of TB is 10-times more than that of the general population, therefore, this segment needs also to be kept in focus. There is an urgent need to undertake a strong national TB program that can coordinate TB eradication activities, better management of latent TB infections especially in migrants from endemic countries, and chalking of effective control and management strategies for diabetics and HIV patients [43].

The highly endemic areas for MDR-TB have been recognized as Central Asia, Eastern Africa, Eastern Asia, Eastern Europe, Central Africa, Northern Europe, South-Eastern Asia, Southern Africa, Southern Asia, Western Africa, and Western Asia [44].

In a study from the Oman region, drug resistant TB was identified as a serious public health concern having association with main risk factors that included previous TB treatment, female gender, and younger age. It was emphasized that public health professionals and clinicians need to be made aware of early detection and right treatment for the right period in order to deal the resistance issues [45]. A significant resurgence of TB has been observed in the industrialized countries in the past two decades that may be attributed to HIV. Moreover, the migratory phenomenon due to poverty, wars, and wealth disparity may also have an influence. Treatments for M/XDR-TB are far from optimal: this is especially true for the migrants living in high-income countries. Migrants have a slow outcome in TB treatment that may be attributed to their inability to communicate, poor understanding of the administrative requirements of health care systems, and fear of being deported if found to be affected with the disease [46].

Pakistan is the sixth-worst TB-affected country in the world. Counselling of TB patients is having a significant impact on the outcome of the disease. The DOTs strategy that in fact has been introduced by the WHO is having a significant impact if the patients are counselled in an effective manner [47].

The TB cases in 2014 from Middle Eastern countries stood at 58,252 with the largest fraction of reports (22%) from Turkey. The notified number of TB cases from Saudi Arabia were 3336 in 2014. The global M/XDR burden was higher than anticipated, but no substantial testing for MDR-TB was carried out, which may have contributed to aggravation of MDR-TB in the Middle Eastern countries and could exacerbate the challenge of controlling the disease. Although the national survey has indicated a low presence of MDR-TB in new (1.8%) and previously treated (15.9%) cases, the figures can rise at any instant. Saudi Arabia is at a global focal point due to the influx and outflux of pilgrims throughout the year, as well as the expatriates who constitute a big portion of the population. Therefore, this region is not only more prone to the national spread, but may become a hotspot for the spread of TB and especially MDR-TB [48]. *M. tuberculosis* is endemic in the Middle East with incidence of 14 and 18 per 100,000 inhabitants in KSA and Bahrain, respectively. Another species identified in this region is *M. riyadhense* that may present with manifestations related to bones, brain, lymph nodes, and pulmonary sites. At present, this species does not respond to standard antituberculosis therapy and thereby results in the development of paradoxical inflammatory syndrome, which may occur after 6–8 weeks of therapy. Although it is a rare disease, in the perspective of Hajj and Umrah its public health relevance needs to be taken care of [49].

At the global stage, US can be regarded as model of health care system where incidence of TB has declined significantly to 3/100,000 that may herald a total elimination of this disease there. The success in US can be attributed to the availability of advanced diagnostics, extensive drug susceptibility testing, and adherence to the set guidelines [50]. However, the global TB burden is at the rise with over 8.7 million new cases and 1.4 million deaths each year, and the magnitude is quite understandable. The resistance in MTB is a serious global issue. The therapeutics include the first line treatment based on four drugs: RIF, INH, ethambutol (EM), and pyrazinamide (PZA), along with streptomycin (SM) in cases where therapy fails with the first four mentioned drugs. In the case of no response to RMP and INH, the strain is termed as MDR. In cases where resistance is extended to any fluoroquinolone (FQ) and a second line parenterally administered drug (like kanamycin, capreomycin), such a scenario is considered to be XDR-TB, a phenomenon that has been observed in nearly 100 countries [51].

Eradication of TB by 2030 is part of SDCs set by the WHO. The top six TB-burdened countries contribute 60% of new cases to global numbers that require stringent governmental policies as well as public health reforms. These highly TB burdened countries also have a major contribution in the global migrant manpower that may contribute to TB dynamics, especially in the case of M/XDR-TB. There is a strong need to define and monitor TB, especially M/XDR-TB niches. The migratory phenomenon is not confined to economic reasons: other factors like poverty, famine, political unrest, and wars may also contribute significantly into the spread of highly communicable diseases along with comorbid conditions, making a huge public health challenge for the nations that host such refugees and too entire global health systems. The risk remains for countries that have eradicated or are on the verge of eradicating particular infectious communicable diseases such as TB, thus facing another public health challenge. The temporary migration in the form of large congregation in religious and social gatherings may also contribute into the gravity of the problem.

## 3. Genetic Aspects of TB

Whole Genome Sequencing (WGS) is useful in the detection of unsuspected outbreaks; therefore, it is a useful research tool and can also be part of surveillance programs to help in the tracking, monitoring, and control of TB. It also aids in the recognition of MTB strains associated with drug resistance and in the identification of hotspots [52]. Co-existence of two or more strains of mycobacteria may present with a complex challenge of misidentification of MTB. Increased genetic understanding of DR-TB will be instrumental in the development of rapid detection diagnostics. However, WGS is not available at all places, and if not carried out then the complete picture is not visible [53].

Atypical MTB strains of Beijing lineage are widespread in South Africa and have shown resistance to up to 13 drugs at multiple occasions [54]. In a Sri Lankan study, the Beijing lineage affected patients who were young with an out-of-country travel history. This is important as far as global TB epidemiology is concerned, which requires policies and practices review of the emigration/immigration at individual countries having high TB burden. It is also important for those countries that have low TB burden but have a great influx of immigrants. There is a need to monitor the kinetics and trends of MTB lineages especially Beijing lineage and others that have well-documented drug resistance in order to devise appropriate actions to combat the associated risks [55].

The Central Asian (CAS) and East African Indian (EAI) lineages of MTB mainly affect the highly populated eastern hemisphere, including the China and India along with 20 other high TB burden countries. The CAS lineage has been reported to be more prevalent in younger patients in comparison to EAI as well as the proportion of M/XDR-TB was also high in the CAS group. Genomic data mining is crucial in order to better comprehend the TB epidemic that is extending its horns due to growing resistance issues. Such studies highlight the ways to better control the TB by defining the specific risks associated with these lineages. In mass gatherings, since people from around the globe get together, any spread of microorganisms may result in the transmission of lineages at a genomic level to other regions, and such studies are vital to determine the dynamics taken by these lineages [56].

The phenotypic and genome-based analyses provide more insights in tuberculosis transmission dynamics. There is a wide array of diagnostic tests capitalized for evaluation of MTB. A summary of different diagnostic techniques for the diagnosis, susceptibility testing, and molecular characterization of MTB, with their advantages and limitations are briefly given in Table 2.

Interestingly, the Saudi population appear to have less of a problem of DR-TB. However, it is pertinent for the health authorities of the Middle Eastern countries, especially Saudi Arabia, to consider more genomic evaluations of *M. tuberculosis* to identify the prevailing lineages as well as the drug resistant genotypes. This will also be of great significance for the public health decision makers to select the most appropriate diagnostics as well as suitable drug regimens for a particular country within the Middle East [57].

Furthermore, genotyping techniques have significance in the public health management of TB. Clustering data are very useful in the development of public health strategies that play pivotal roles in reduction of transmission as well as reactivation of MTB. Beijing strains constitute a growing proportion of total cases and are reported from high incidence settings including China, India, Saudi Arabia, Russia, Indonesia, Uzbekistan, Turkmenistan, South Africa, Bangladesh, Vietnam, Spain, Taiwan, and Japan. Beijing lineage presence in younger age groups reflects recent transmission. Beijing strains that were MDR were also universally found to be resistant to SM. High genotype diversity can be seen in low-incidence settings, reflective of migration to a particular region from around the world rather than local transmission. In this situation epidemiological studies of the MTB lineages are important [58].

The WGS studies are instrumental in the design of diagnostic tools for effective disease controls. In the Philippines, the most prevalent MTB lineage is the Manilla ancient linage 1 strain, followed by lineage 4 (European–American), and the East Asian strain. A high proportion of the strains were multidrug resistant with some MDR-TB isolates having near similar genomic variation indicating transmission [59].

Compared to WGS, the spoligotyping data arise from the presence or absence of 43 spacers that are reduced to an octal code to compare with SITVITWEB, which is an international data base. Beijing lineage is typically characterized by the absence of 34 initial and by the presence of 35–43 spacer oligonucleotides. It has been reported from around the world; however, it is less frequent in certain parts of South America and northern- and central Europe. This lineage has reported association with drug resistance and it has the ability to acquire drug resistance and disease transmissibility. SIT1 is prominent in Saudi Arabia. SIT190 has been found to associated with XDR-TB. This lineage has also been reported for RIF, INH, STR, PZA, and linezolid resistance. The non-Beijing lineages include the Central Asian (CAS) and East-African Indian (EAI) linages. These lineages have also been reported for their varying degrees of drug resistance. In a study from Saudi Arabia EAI lineage strains were found associated with MDR. Genetic polymorphism is an essential feature within all lineages of MTB. Although high resistance rates may not be associated completely with the prevalent lineages, some association may be attributed to drug resistance [60].

Genetic diversity has a significant influence on disease outcome, virulence, severity of disease, and host immune regulation. Particular MTB strains acquire drug resistance due to their higher ability to mutate. It has also been found that MTB Beijing (lineage 2) strains exhibit higher mutation rates compared to lineage 3 strains in the case of RIF resistance. The lineages 2 and 4 have also been related to higher virulence. Highly virulent strains have more chance of exposure to antibiotics since they are not removed by the immune system. MTB has also been found to grow near the interface of air–fluid cavities in a biofilm-like formation. This situation also favors a high mutation rate that consequently may favor the rise of drug-resistant mutants. This can also be the case among the previously treated patients. The biofilm phenotype is highly variable among the MTB strains (thick biofilm producer to non-producer). The prime component of the biofilms is methoxymycolic acid of which production is low in lineage 1. From this perspective, the addition of an antibiofilm molecules with current anti-TB drugs may be a good strategy. MTB survival mechanisms in the course of drug therapy are governed through a diverse set of mechanisms. Mutations resulting in drug resistance are preceded by non-specific mechanisms like modulation of cell wall structure, expression of specific efflux pumps, and biofilm formation. Since the host immune response is of vital significance, optimum TB treatment should be personalized, keeping in view patient and the genetic diversity of bacteria that causing the infection. Thus, host-directed therapy with an antibacterial response to anti-TB drugs will be significant in the outcome of TB treatment [61].

**Figure 1:**
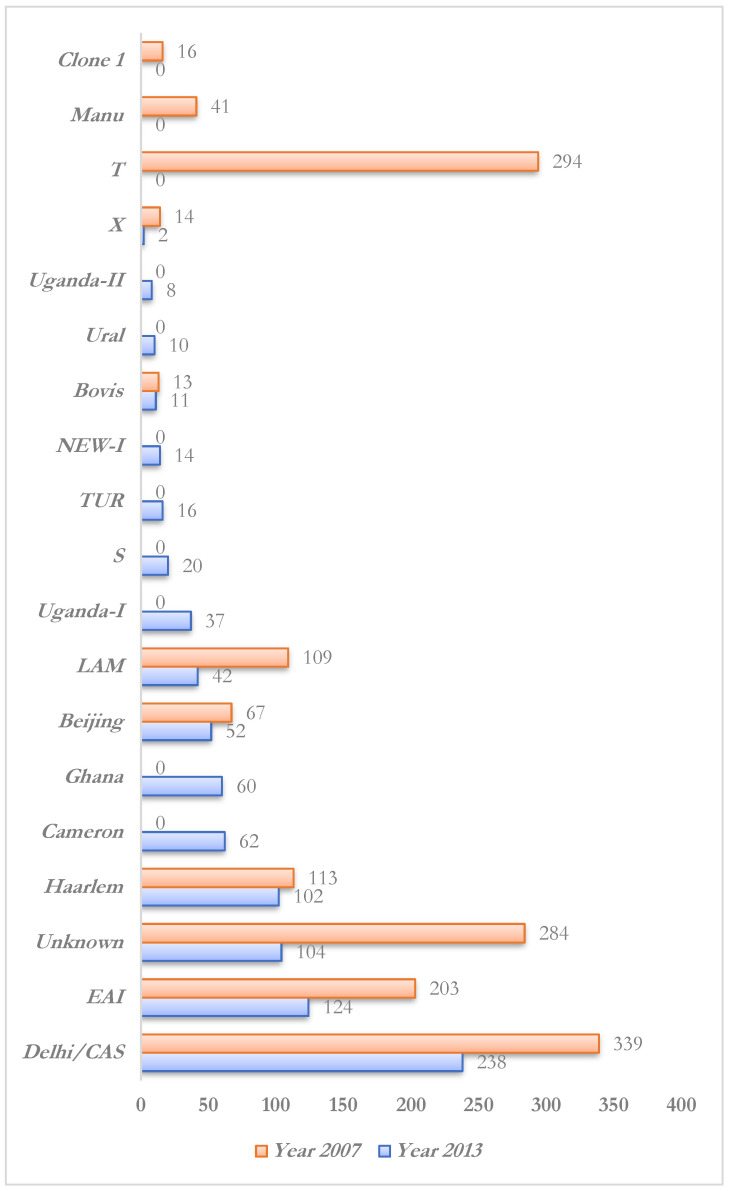
A comparison of phylogenetic diversity of *M. tuberculosis* isolates from KSA (Data from Al-Hajoj et al., 2007 [62] and Al-Hajoj et al., 2013 [63] (CAS = Central Asian; EAI = East African Indian; LAM = Latin American–Mediterranean).

As far as the genomic MTB studies from KSA are concerned, unfortunately little literature is available. For instance, the first study of such kind was carried out in 2007 by Al-Hajoj et al. [62], showing almost 81% MTB isolates belonging to recognized phylogeographic clades. It included the 22.5% CAS, 19.5% ill-defined T clade, 13.5% EAI, 7.5% Haarlem, 7.2% LAM, 4.4% Beijing, 2.7% Manu, and 0.9% each for X and Bovis lineages. Two clonal complexes were found to have exclusive KSA spoligotyping signatures that were also shown in EAI and CAS clades at the secondary level typing (Figure 1). The same research lead in 2013 reported [63] even higher phylogenetic diversity in MTB (Figure 1) as nearly all recognized genetic lineages showed their presence in younger patients with 26.4% CAS/Delhi, 13.7% EAI, and 11.3% Haarlem lineages. The advanced aged patients were found to have preponderance of S, TUR, Ghana, and Uganda—I lineages. Major clustering was observed in cases from the Haarlem, Beijing, and LAM lineages. This all indicated a very high diversity in MTB strains from KSA, having substantial associations with transmission dynamics, demographics, and patients’ origin. It also indicated a continuing transformation in the prevalence of strains in the country. It is important to note that similar studies with larger population sizes will give a clearer picture of the status of endemic strains of KSA.

Global DR-TB is on the rise, requiring extensive treatment (2 years or longer) along with potentially toxic drugs and poor outcomes (about 50% DR-TB patients die due to it). The second line drugs are expensive. All the said reasons necessitate the prevention of a DR-TB crisis. The key components to tackle the scenario include prevention of the development of resistance in drug susceptible TB through provision of quality treatment, rapid testing and detection of DR-TB, provision of effective treatment and care, and taking steps to minimize transmission. Developing countries have put TB at a low priority which may result in a global health emergency [64].

The design of new diagnostic tests and investigation of resistance rely heavily on genome-wide study, in particular for TB, which is finding growing resistance. Sampling for such studies should be systematic and resistant isolates be evenly distributed across collection sites. Maximum coverage of all the lineages of MTB and the regional variations needs to be considered in order to fully characterize genotype–phenotype relationships. Better understanding of genetics of antitubercular phenotypic drug resistance will facilitate the development of accurate molecular diagnostics for resistant MTB strains [65]. Though WGS has a revolutionizing power in drug susceptibility tests, the associated costs, facility requirements, and other issues need to be addressed prior to its use [66].

The use of genetic tools, especially WGS, has proven useful even in unsuspected outbreaks. They are of value not only in the identification of resistant strains but also in the markup of hotspots, which are important from a public health perspective. Molecular research also has a key influence on detection and diagnosis; thus, it needs to be implemented in all reported regions and should be made mandatory for the endemic zones especially where M/XDR-TB is persistently reported. Cost is the main obstacle that needs global assistance from bodies like the WHO at an international level, and from concerned governments at their respective national levels. The choice of different types and forms of genome-based studies lies with regional authorities based on their financial implications. However, at least a standard genomic tool must be adopted: if not in all regions, then in particular regions with TB and especially M/XDR-TB prevalence.

## 4. Resistance Issues with Antitubercular Drugs

DR-TB has been recognized by the WHO as threat to global health security that requires tools and services at a broad perspective for quick diagnosis, treatment, and care. Treatment of DR-TB is a complicated task requiring multidimensional approaches. Maximum capitalization of advanced diagnostics require the removal of the patient and a provider of level barriers [67]. Although there is a significant development in cutting off the diagnosis time and initiation of treatment using rapid diagnostic methods, the removal of all delays to diagnosis is not possible due to other hurdles. The advent of rapid molecular diagnostics has improved the routine drug susceptibility evaluation in many countries. The involvement of sequencing in surveillance campaigns removes the limitations of phenotypic characterizations and gives additional support to molecular epidemiology.

The XDR-TB and MDR-TB are prime public health concerns. People displaced from endemic regions and residing in substandard, undernourished conditions who have no access to medications can be a very easy target for such microbes. Such patients serve as a reservoir and can very easily transmit the disease to the general public, and to health care providers [68]. An estimated 0.55 million new MDR-TB cases occurred during 2018 and only a fraction was diagnosed. These cases increase alarmingly each year [69].

In a Chinese study, bedaquiline was found to significantly reduce DR-TB. The use of bedaquiline-containing regimens was found to prospectively reduce the incidence, prevalence, and mortality of DR-TB. Similar prospective modelling studies are required to devise strategy against DR-TB in other countries [70]. However, the emergence of bedaquiline resistance from northern India through a novel non-synonymous single nucleotide variation (SNV) G162E in Rv0678 gene is alarming. Such reports necessitate the detection of resistant strains for prompt treatment and the design of rapid methods for their detection. Since novel SNVs have direct impact on the drug resistance, region-specific probes would be required for effective diagnosis and treatment of resistant TB cases [71] that demand genome-based studies. The challenge of TB resistance is immense: even the newly inducted molecules are facing resistance. Host-directed therapy (HDT) may offer a long-term solution in the treatment of M/XDR-TB. The HDTs need to be trialed against the resistant cases [72].

In a first molecular epidemiology report, multiple clusters of local transmission of MDR-TB from Kuwait were identified which had escaped the routine surveillance. Rapid molecular diagnostics that focusing on hot spot regions of katG315+inhA-RR and rpoB yielded a sensitivity of 98% for RIF and INH resistances, and 96% for MDR-TB. The MDR-TB strains (57%) were also found to have pncA mutations thus were PZA resistant [73]. The report of 29.5% monoresistance and 3.3% MDR was from Tabuk in the northern region of KSA. GenoType MTBDRplus assay was used to determine the mutations in rpoB, inhA, and katG genes for the INH (26.2%) and RIF (3.3%) resistances [41]. RIF resistant TB was found to be higher in HIV seropositive patients in an Ethiopian study. Similar resistance among previously treated patients was comparable to other regional countries. Low socioeconomic status and long-distance travel to get access to a health care facility were major factors for non-adherence to treatment [74].

In a German study, drug-resistance patterns were significantly complex, signifying the need for detailed drug testing prior to therapy. Increased INH resistance suggests INH-based preventive therapy should not be used for latent TB infections. The highest numbers of drug-resistant cases were observed for the countries with high TB burden. High drug-resistance was observed for cases who were foreign-born. Prior TB cases had a significantly higher proportion of resistance. Children, females, foreign-born, and having pulmonary TB were found to increase the risk of acquiring drug resistance. Rapid drug susceptibility testing (DST) was suggested for patients with a high risk of drug resistance prior to the phenotypic tests. Demographic characteristics need to be kept in view as well as the full drug resistance profile to optimize treatment outcomes [75].

As per WHO estimates, 10 million TB incident and 1.6 million TB deaths were reported in 2017. With about 3.5% new TB cases, 18% previously treated cases were MDR-TB, with 8.5% being the XDR, situation is alarming. In 2018, WHO reclassified TB drugs for longer conventional regimen for MDR-TB. In that grouping, the first group included levofloxacin/moxifloxacin, bedaquiline and linezolid; the second group included clofazimine and terizidone/cycloserine. Finally, the group third included PZA, imipenem-cilastatin, meropenem, ethambutol, delamanid, amkiacin/SM, theionamide/prothionamide, and p-aminosalicylic acid, which are used in cases where the first two groups are not usable [66].

Line probe assay (LPA) was reported to be more effective in the detection of TB and MDR-TB, being rapid, reliable, and efficient for determining TB and the resistance pattern instrumental for early diagnosis and establishing suitable therapy. High prevalence of inhA was also signified through LPA [76].

In an Ethiopian study, the median recovery time of MDR-TB patients was 21 months with close monitoring of patients’ adherence to treatment. Factors like distant location, scarcity of health facilities, and low economic- and social status were highlighted as the main hurdles. Government was urged to rectify the issues in order to reduce the recovery time of MDR-TB patients with measures like better patient counselling and support and use of digital tools for surveillance of treatment adherence. Furthermore, rapid and early detection with proper treatment of drug susceptible MDR-TB was envisaged to lower the recovery time as per WHO guidelines [77].

The good performance reports for Xpert^®^ MTB/RIF used in the evaluation of resistance in tuberculosis justify its further implementation. Sensitivities ranging between 58 to 100% have been reported for Xpert^®^ compared to culture sensitivity assays [78].

Substantial evidence has been found regarding tobacco smoking that increased the risk of DR-TB. However, tobacco smoking was shown to be an independent risk factor for DR-TB. Additionally, TB patients may benefit from smoking cessation as their risk of developing MDR-TB, XDR-TB is lowered. Thus, interventions should be made to encourage TB patients to quit smoking as soon as they are diagnosed [79].

Reports of total drug resistant (TDR) TB (>30 cases) in countries like India, Iran, Italy, and South Africa are very alarming. It is important since a good number of pilgrims come from Iran and India, as well as a migrant force from the latter; therefore, the countries where such reports appear need to be closely monitored and additional testing needs to be recommended [80].

The HIV-seropositive patients with TB have shown high rates of RIF resistance. This is of particular concern in the MDR and XDR-TB scenarios that are posing a huge challenge to TB control programs especially in developing countries [81].

Reports of extra-pulmonary XDR tuberculosis are challenging especially in resource-limited countries. Thus, rapid diagnostics need to be available with the implementation of strict preventive strategies. Risk to health professionals from resistant mycobacteria also looms since they are the frontline force to treat infected patients [82].

The resistance against antitubercular drugs is established and M/XDR-TB is a prime public health concern. Rising resistance against newly approved drugs is of particular concern to the health and public health authorities. The judicious use of ATT in accordance to well defined standard operating procedures (SOPs) alongside strict monitoring is the only way to tackle this serious issue. Drug resistance patterns are complex thus necessitating detailed drug testing prior to therapy. It also requires keeping track of demographic characteristics along with full drug resistance profiles to achieve optimum treatment outcomes. Reliable standard detection and sensitivity assays are pivotal in the success of ATT therapeutics. Moreover, independent risk factors like smoking found to be associated with DR-TB need to be kept in consideration while framing a public health policy meant for the addressal of DR-TB and TB. The most significant current public health concern is the TDR-TB reports from certain regions of the world. They need extraordinary surveillance. Moreover, similar surveillance is required for patients with HIV comorbidity with TB. In this whole scenario the biggest challenge and concern is the risk to frontline health care professionals’ of exposure to the resistant mycobacteria, which needs additional tiers in healthcare policy making and implementation for such health care workers (HCWs).

## 5. Tuberculosis in KSA

In a Saudi study conducted at a TB ward of a tertiary care hospital, the extra-pulmonary TB isolates showed higher resistance for first line TB drugs with nearly a quarter (27%) of all clinical isolates found resistant to INH, RIF, and EM. The isolates from female patients were found to be more resistant to EM, while the extra-pulmonary cultures showed a higher resistance against INH [83].

A pattern similar to global prevalence of tuberculosis drug resistance was observed at a tertiary care hospital. Mono-resistant strains were 60.8%, multidrug resistant strains were 25.49%, while 13.73% were polyresistant. The drug resistance was found to be more prevalent in non-Saudi nationals. The spread of drug resistant organisms is more likely to happen in the months of Hajj and Ramadan since they are major religious periods [84]. Yet in another Saudi study, the MDR-TB isolates were shown to be of the Haarlem Family which has global distribution. Moreover, in a national study on molecular epidemiology of TB in KSA, a mixed phylogenetic distribution of MDR-TB was observed in which Beijing lineages had a significant association with MDR-TB. In an earlier study from KSA, no specific distribution of MTB phylogeographic lineages in the indigenous and the expatriate populations was reported which may reflect transmission among these two groups. Furthermore, the extensive trade and other related contacts of this region with the rest of the world is a crucial factor. All this needs further rigorous monitoring and stringent adherence to national TB control policies [85]. A Saudi national survey on antitubercular drug resistance showed 1.6% MDR-TB [86]. The report showed 29.5% monoresistance and 3.3% MDR from the Tabuk present in the northern region. GenoType MTBDRplus assay was used to determine the mutations in rpoB, inhA, and katG genes for the INH (26.2%) and RIF (3.3%) resistances [41,87].

In a recent Saudi study, the mortality rate of TB was found to be 22/1000 person-years. It was found to be high in males, the elderly, and those having other comorbidities. Monoresistance, RIF resistance, MDR-TB, and XDR-TB were also reported. A recommendation was made for the implementation of an efficient TB diagnostics [87].

Implementation and adoption of DOTs in the KSA took place in 2000 at the facility level and later in 2013 at the community level. No significant difference was observed at the community level before and after the implementation of DOTs. The main administrative factors that were responsible for the impact on program performance were identified, such as lack of policies, procedures, and strategic planning. In order to achieve TB elimination, policy updating, expansion of laboratory net, implementation of quality measures for laboratories, along with endurances of all required financial resources was recommended [88]. In this regard, community mobilization of mobile teams served as an effective manner to improve TB treatment and follow-up, reduce mortality, and improve treatment outcomes. KSA has many challenges in the control of TB, including a huge number of foreign workers especially from the TB endemic countries, illegal immigrants, and religious visitors. The mobile teams consisted of a physician, nurse, health inspector, and a driver. The prime objectives were to ensure the adherence to DOTs therapy, focus on the treatment and sputum follow-up, detection of cases through contact tracing, and visiting private hospitals to look for signs of TB infection. This service worked well with significant improvement in the treatment outcome (treatment success of 92%), decreased mortality of 1.18% compared to 9.31% of non-mobile patients, and with a failure rate of only 0.86%, which all signify the importance of the mobile teams. Following the success, this model can be adopted for the entire KSA, through proper planning [89]. The overall default rate of 3% in patients served by the mobile outreach team compared to those who did not receive such service (22%) is quite low. Compliance scores were also significantly higher in the mobile team in serving patients after a 3-month treatment. Thus, mobile outreach DOTs can deliver better understanding of the disease as well as treatment to TB patients. Since this new strategy has worked well in the dissemination of disease information and has yielded better patient compliance, its replication was suggested for other cities of KSA [90]. Saudi Arabia is facing multiple challenges that may cause the rise and spread of MDR bacteria. Recommended actions in this context include strict surveillance of antimicrobial resistance at hospital, regional, and national levels. Furthermore, better infection and prevention control strategies need to be worked and implemented including awareness regarding resistance, so that the inappropriate use of antimicrobials is controlled [91]. TB is more prevalent in the Makkah region, affecting Saudis and non-Saudis equally. The annual Hajj congregation and the Umrah rituals are associated with infectious diseases, especially respiratory tract infections. The past data of seriously ill pilgrims with TB suggest a large number of undiagnosed patients that may play a significant role in transmission to other pilgrims. The main limiting factors in defining the burden of TB include mobility of pilgrims, difficulty in sample collection, storage, and availability of rapid testing for ruling out MDR-TB. There are no proactive specific recommendations for detection or prevention of TB in pilgrims or KSA residents. Despite limitations in evaluation of mass gatherings for diseases like TB, opportunities are there to conduct studies in order to get accurate data on TB burden and transmission risk, especially from a global perspective [92]. The TB surveillance data in Al-Madinah province in 2011 were found to be inconsistent due to problems in recording and record systems. There were issues identified with respect to patient contacts and a significant amount of under- or nonreporting was also observed [93].

GHC has realized that due to overlaps in patient care between the states, whole genome sequencing can be of significant worth in the identification of emerging pathogens as well as helping in the investigation of inter- or intrastate outbreak; therefore, a callout has been made for the use of WGS for antimicrobial resistance (AMR) in the GHC states. A state-of-the-art WGS service is operationalized in the King Abdullah International Medical Research Centre, Riyadh, KSA. The facilities need to be extended for research on resistance issues in MTB [94]. The phenotypic and genome-based analyses provide more insights in tuberculosis transmission dynamics. Interestingly, the Saudi population appear to have less of a problem with DR-TB. However, it is pertinent for the health authorities of Middle Eastern countries, especially the KSA, to consider more genomic evaluations of *M. tuberculosis* to identify the prevailing lineages and the DR genotypes. This will also be of significance for the public health decision makers to select the most appropriate diagnostics and suitable drug regimens for a particular country within the Middle East ambit [57].

The national TB data from KSA suggest the rate of MDR-TB to be in equivalence with the WHO’s reported rate, which is comparatively high compared to western countries. The MDR-TB cases were reported more in females, younger age groups, and previously treated TB patients. The western region of KSA reported higher prevalence of MDR-TB compared to all other regions, mainly due to the influx of pilgrims from around the world to the holy cities of Makkah and Al Madina [49].

KSA is suffering from MDR-MTB transmission as predicted by the high cluster rates and similar resistance-conferring mutations. It is alarming and requires interruption through better surveillance, diagnosis, and therapy. In a 10-year drug-resistance study of a tertiary care hospital of Jeddah, KSA, high involvement of young people was revealed. Elevated levels of resistance to first line drugs were also seen [95]. Younger people of the KSA have been found to be at risk. In Middle Eastern countries recent transmission and reactivation cases are present, but the latter have an increasingly significant role [96]. Risk factors include drug abuse, alcohol consumption, being imprisoned, homelessness, HIV infection, and being an immigrant. TB transmission in immigrants is high since they live in small and crowded places with inadequate facilities and poor sanitation, face stress, and are malnourished. The MDR ratio in the Al Madina province was similar to the other regions of the KSA. The prevalence of TB was higher in Saudi compared to non-Saudi residents. Children were also found to be MDR carriers, thus strict surveillance is required [97]. It is evident that KSA is facing a considerable challenge of TB resistance, and new challenges are emerging with cases of fluoroquinolone and aminoglycoside resistances. The presence of a pan-resistant situation is alarming and signals the appearance of XDR-TB in near future [98]. The TB cases in 2014 from Middle Eastern countries stood at 58,252 with the majority of reports from Turkey with a 22% share. The notified number of TB cases from KSA were 3336 in 2014. The global MDR/XDR burden is higher than anticipated, but no substantial testing for MDR-TB was carried out, which may contribute to aggravation of MDR-TB in Middle East countries and can be a greater challenge in the control of disease. Although the national survey has indicated a low presence of MDR-TB in new (1.8%) and previously treated (15.9%) cases, the figures can rise at any instant. The KSA is at global focal point due to the influx and outflux of pilgrims throughout the year, as well as the expatriates who constitute a big portion of the population. Therefore, this region is more prone to the national spread and may become a hotspot for the spread of TB and especially the MDR-TB [48]. The incidence of TB in Saudi Arabia is 14 per 100,000 population. The country has the highest prevalence of MDR-TB in the northern and southern regions, which were followed by the western and central parts with eastern areas being the lowest impact [99].

The WGS based approach has the advantage of high resolution and gives insight into detailed resistance for all the drugs, which can be a vital step for future health care planning of the KSA as it facilitates accurate diagnosis, excellent management of cases, and better understanding of the transmission dynamics; these features are instrumental in the settings that encounter high mobility rates and immigration [100].

In this context, the role of antibiotic stewardship programs (ASPs) in the reduction of resistance and appropriate antimicrobials use is well-established. Its implementation in the Gulf Coordination Council countries is below par. Keeping in view the rise in MDR-TB, prompt ASPs need to be implemented at the local health care level and at strategic focal points to be an integral part of the national surveillance program [101].

In an interesting study from KSA, *M. tuberculosis*, *M. bovis*, *M. caprae,* and *M. microti* have been reported from camelids. TB has also zoonotic potential and risk of bovine TB, especially in handlers, is of special public health concern. The prime causative agent of bovine TB is *M. bovis*, which can be transmitted to humans through ingestion of non-pasteurized milk and dairy products or through respiratory droplets while in proximity to infected animals. *M. bovis* has been isolated from camel milk which is commonly used in raw form thus making it a potential source of TB infection. Surveillance of animals and health safety precautions should be adopted to avoid associated risks [102].

Earlier there had been a disparity and inconsistency observed among general physicians, resident physicians, and specialists regarding the rational use of antimicrobials in the KSA, thus an irrational and excessive use of antimicrobials was observed. The situation required educational interventions to improve the rational use of antimicrobials and reduce the consequent resistance issues. Furthermore, rigorous and strict regulatory steps alongside a futuristic policy framing to counter the irrational and overuse of antimicrobials was sought [103].

*M. tuberculosis* is endemic in the Middle East with incidence of 14 and 18 per 100,000 population in KSA and Bahrain, respectively. Another species identified in this region is *M. riyadhense,* which may present with manifestations related to bones, brain, lymph nodes, and pulmonary sites. This species may not at times respond to standard antituberculosis therapy and thereby results in the development of paradoxical inflammatory syndrome, which may occur after 6–8 weeks of therapy. Although it is a rare disease, in the perspective of Hajj and Umrah gatherings, its public health relevance needs to be taken into account [49].

Current molecular epidemiological data from KSA suggests a mixed phylogenetic distribution in MDR-TB, indicative of an influx of organisms across the globe due to trade and other migratory activities. Adherence to established guidelines related to TB is a key feature of KSA health policy but the current shift in TB and its resistance dynamics requires further strict actions in policy framing and in public health quarters. The mobile outreach team model application has yielded remarkable results thus making it a good example to follow for better tackling the situation. However, it needs implementation across the KSA and specifically the major pilgrim and migrant influx cities like Makkah, Al Madina, and Jeddah to curb and halt the disease to the other parts of the country and to other regions and countries. Since the likelihood of KSA turning into a global TB hotspot is very high, such steps are of prime importance and are already implemented, but need constant revision in shifting global TB dynamics. It is pertinent to mention that MTB and other mycobacteria have zoonotic potential, and this aspect needs to be highlighted specifically to the public health concerned quarters as the resistance phenomenon may also take the zoonotic course for its spread. Another specific segment is the implementation of ASPs at local health care levels, at the strategic focal points, and as part of national surveillance programs. Such program implementation requires healthcare professionals be on the same level in order to improve the rational use of antimicrobials and reduce the resistance issues.

## 6. Impact of Treatment Options

The timely initiation of TB drug therapy is of vital significance. In this respect, logistic management is important to ensure a 24 h Xpert “On the spot strategy” for the timely detection of TB, to initiate of appropriate therapy, and decrease TB-associated mortality [104]. This is the case of simultaneous use of GeneXpert and MTBDRplus for RIF/INH, FQs, and second line injectable anti-TB drugs to screen XDR-TB in Sudan. More care was suggested for MDR-TB patients that have increased mortality and default. It is also essential to supply drugs to MDR-TB patients for their entire treatment period [105]. Moxifloxacin can be considered for pediatric patients with TB, but needs further prospective studies [106].

In this regard an important consideration is the pattern of drug metabolism in certain TB drugs such as INH, which has variation in the rate of acetylation in TB patients, which may impact the outcomes of therapy. This is important as the sputum conversion failure is two times higher in the fast acetylators compared to slow acetylators [107].

The use of Xpert^®^ MTB/RIF is increasing due to its rapidity and sensitivity. It is helpful in the detection of smear negative cases. Airborne infection isolation (AII) room facilities are required for the hospitalization of presumptive TB patients. Such a room is for a single patient and has a negative-pressure ventilation system. HCWs attending an AII room have to follow protection protocols. Isolation in such a room is discontinued if three smears collected at intervals of 8–24 h are negative. Since AII facilities are not available at all hospitals and are scarce and costly, a quick decision is crucial: nosocomial infections, and more alarmingly MDR-TB, make this decision vital. In light of COVID-19, such facilities need to be reassessed and the decision to keep a patient in such an area should be justified [108].

As a special case, a TB patient with severe sepsis can be administered with the empiric antituberculosis treatment. The WHO guidelines also support this strategy, calling for immediate instead of delayed empiric therapy in areas of high tuberculosis and HIV prevalence [109].

As per WHO estimates, 10 million TB incidents with a 1.6 million TB deaths took place in 2017. With about 3.5% new TB cases, 18% previously treated cases were MDR/RR-TB, with 8.5% being the XDR, the situation is alarming. In 2018, the WHO reclassified TB drugs for longer conventional regimen for MDR-TB. In that grouping, the first group (priority) includes levofloxacin/moxifloxacin, bedaquiline, and linezolid and the second group include clofazimine and terizidone/cycloserine. Finally, the third group includes PZA, impipenem-cilastatin, meropenem, ethambutol, delamanid, amkiacin/SM, theionamide/prothionamide, and p-aminosalicylic acid, which are used in cases where the first two groups are not usable. Ageing population vulnerability poses a public health challenge, especially those coming from many Asian countries [66].

The molecular methods for the susceptibility evaluation of MTB are of vital significance due to rapid diagnosis. Furthermore, they are of significance in patients who have some limitations with respect to sample collection [110]. The detection and susceptibility techniques do have a major influence on therapeutics. GenerXpert is a novel real-time PCR assay endorsed by the WHO for diagnosis of TB with evaluation of RIF susceptibility. It provides timely results with good specificity and sensitivity [111]. With minimum biohazards, Gene Xpert^®^ MTB/RIF was found better in the assessment of DST. Due to its ability to shorten the diagnosis of MDR-TB to just 2 h, instead of weeks, it takes on the conventional methods [112]. In this respect, a study from Kosovo found GeneXpert to be able to detect additional cases of TB compared to LJ culturing and smear microscopy. Moreover, it has the added advantages of defining the RIF resistance and eliminating false negative cases [113]. The MTBDRplus assay can serve well in the diagnosis and therapeutic management of Intra ocular TB (IOTB). The recovery of MTB from IOTB samples is relatively low compared to other molecular assays. GenerXpert has an advantage over MTBDRplus due to its automation which reduces manual errors and contamination, but its confining limitation is that it only detects RIF resistance while the MTBDRplus has the ability to detect the RIF and INH resistance [114]. The use of gene Xpert^®^ MTB/RIF has shown good sensitivity, specificity, and accuracy in an Egyptian study which was thus recommended for evaluation of RIF resistance with recommendation of re-standardization of reference in order to obtain more valid results [115]. However, in cases of nonavailability of molecular facilities, better alternatives should be planned. In this respect, direct 2,3,5-triphenyl tetrazolium chloride assay can be used as an economical alternative method for its rapid and accurate detection of RIF and INH resistance when the molecular methods are not available [116].

There is good evidence that tobacco smoking increases the risk of DR-TB. However, it is an independent risk factor for DR-TB. Pooled risk of MDR-TB was 1.45 times higher in smokers than non-smokers. Additionally, TB patients may benefit from smoking cessation as their risk of developing MDR-TB, XDR-TB is lowered. Thus, interventions should be made to encourage the TB patients to quit smoking as soon as they are diagnosed [79].

Pyrizinamide has an excellent sterilizing activity and shows bactericidal activity in acidic environments. If there is added to triple regimen of INH, RIF, and EM during the first 2 months, it shortens the treatment time to 6 months from 9 months. It also has a synergistic effect with bedaquiline and pretomanid. Keeping in view these potentials, it can be made part of novel treatment regimens meant to shorten TB treatment in future [117]. Although pyrizinamide is an important anti-TB drug that is effective against the non-replicating MTB, it is also experiencing resistance, especially in the pncA [118].

Fluoroquinolones are useful chemotherapeutic agents that can be used in conjunction with other TB drugs for the management of MDR-TB cases. The increased use of FQs has resulted in FQ resistance (ofloxacin resistance stood at 62.3% in previously treated TB patients) in MTB, which is a defining feature of XDR-TB. There is an urgent need for accurate and quick molecular testing for the detection of pre-XDR-TB in order to manage it in time [119]. MDR-TB rates are higher in patients with previously incomplete anti-tuberculous treatment. Such patients pose a severe threat to transmission in the community. Therefore, molecular characterization and patient-specific resistance profiling is important [120].

The use of antitubercular treatment in patients with TB uveitis has low treatment failure compared to those patients in which choroidal involvement and vitreous haze phenomena is there [16]. Such clinical implications must be kept in mind prior to the use of an ATT.

TB was declared a global emergency in 1993 by the WHO. The BCG vaccine is only effective in children and not in adults, therefore, there is need for effective TB vaccines [121]. However, protection provided by the BCG vaccine appears to vary on geographical and strain bases [122]. Supplementation of vitamin D in TB patients enhances sputum smear and the conversion of cultures. It is speculated that such supplementation improves the disinfecting activity of macrophages thus increases the sputum conversion [123]. Thus its incorporation into the ATT be made and continued for 200 days [124].

Tuberculosis is more common in immigrants from Asia. Drug induced hepatotoxicity is common in tuberculosis patients; therefore, biweekly liver function testing is recommended following the initiation of antitubercular treatment in the first month, thereafter, monthly follow up until the completion of therapy [125].

On time initiation of ATT is of great significance in effective TB treatment. Correct diagnosis plays a major role in planning the right treatment. Drug related considerations like pharmacokinetics need to be kept in consideration in order to make optimum use of therapeutics. The use of justifiable empirical therapy can be done in special circumstances requiring immediate intervention. The option must be logically and rationally utilized along with judicious use of all complementary and alternative medicines to better achieve treatment outcomes. Furthermore, the consequences of ATT following initiation need to be monitored through set protocols to better deal with the side effects and to avoid adverse reactions associated with the chosen ATT.

## 7. Role of Travelling and Mass Aggregations

International travelling was on the rise till 2019 when 1.4 billion travelers [126] moved to various destinations around the world. Although this trend halted due COVID-19-related lockdowns and restrictions on international travel, it will gain momentum as soon as the pandemic subsides. This is an important factor regarding the spread of infectious diseases like TB. Similarly, airborne transmitted diseases remain a main cause of mortality after cardiovascular diseases. TB is a communicable disease that has a high transmission risk.

**Figure 2:**
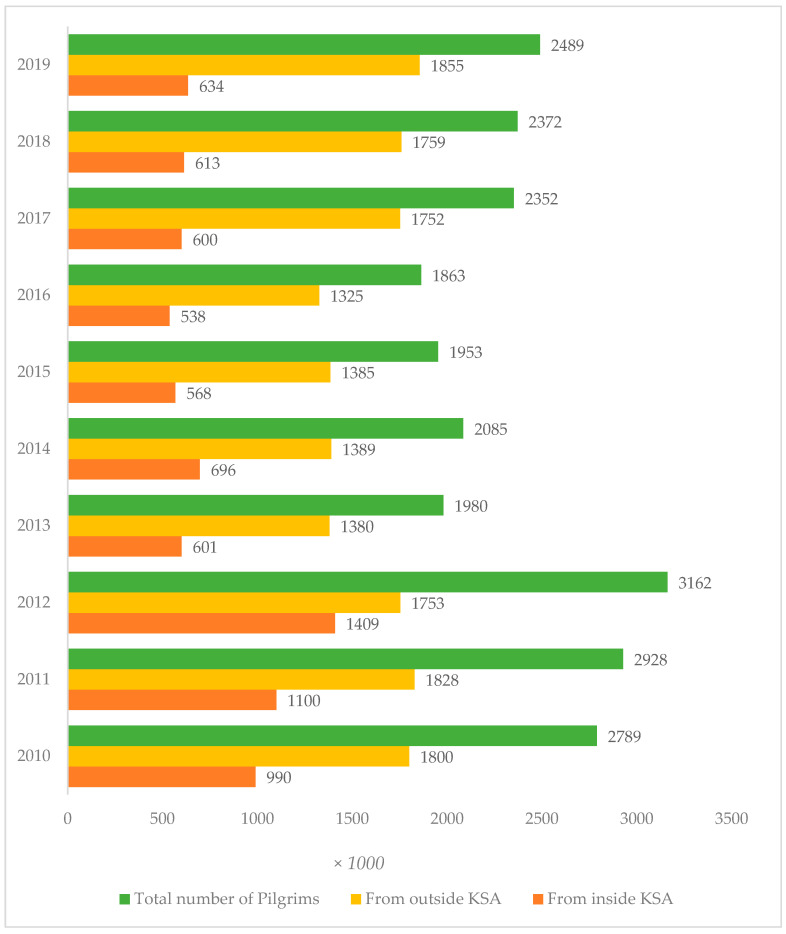
Ten years (2010–2019) Number of International and local Hajj Pilgrims (Adapted from GAStat Haj Reports [128]).

**Figure 3:**
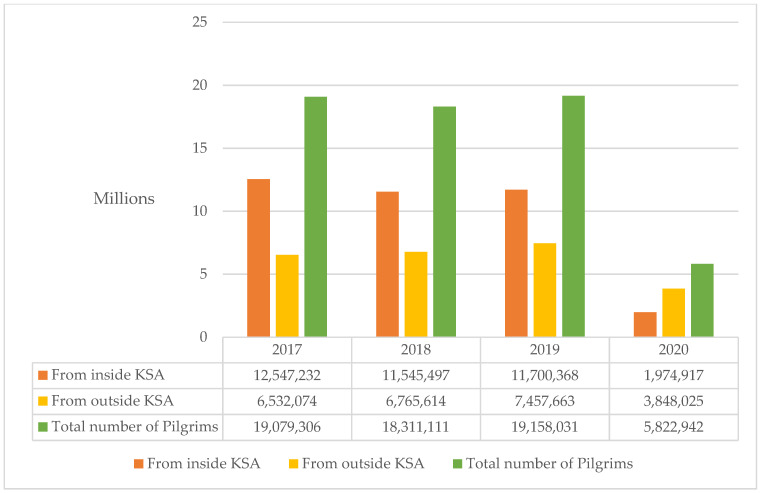
Four years (2010–2019) Number of International and local Umrah Pilgrims (Adapted from GAStat Umrah survey [129]).

Mass gatherings such Hajj and Umrah around the world facilitate the transmission of diseases of epidemic and pandemic potential. There is need for multistage collaborations, extensive surveillance, and increased testing at reference laboratories [127]. The huge number of participants (pilgrims) in both sacred Muslim congregations come in very close proximity, thereby, generating enormous risk of viral diseases and contagious bacterial diseases such as TB. In this connection, the Hajj (Figure 2) [128] and Umrah (Figure 3) [129] pilgrim numbers indicate a persistent increasing trend. Pilgrims from around the world as well as inside KSA flock toward the holy cities of Makkah and Al Madina.

Conventions, large religious gatherings, and similar aggregations pose huge challenge for diagnosis, management, prevention, and control of infectious diseases. This is of concern in the case of TB, keeping in view the endemic status of the countries where such large gatherings take place [130]. Respiratory tract infections are highly prevalent and transmissible at a global level during the Hajj congregations. This results in high morbidity, which challenges health care facilities and puts huge economical stress on health systems. It was also suggested that patients should be educated on performing Hajj at a younger age, avoid self-medication in case of infectious diseases, and keep a complete record of co-morbid conditions [131].

Moreover, pilgrims need to be extensively medically examined with an emphasis on their medical history, vaccinations, education on protective and preventive measures, and chemoprophylaxis [132]. The risk of contracting TB during travel is small, but increases with the length, mode, and destination of travel. In the case of return travelers who have persistence of pulmonary symptoms, TB should be included in the differential diagnosis list with proper investigation. Since the Hajj and Umrah pilgrims come from around the globe, persistence of pulmonary symptoms must be dealt in accordance to criteria set for TB [133]. In a prospective cross-sectional study on Hajj pilgrims, a portion were found to have undiagnosed and untreated active TB, which can be an important source of transmission. Such studies are required from to draft public health policies and plan direct interventions for the optimal surveillance, screening, treatment, control, management, and awareness during Hajj and similar mass gatherings around the world [134]. Over 4 million pilgrims visit the holy cities of Makkah and Madinah throughout the year which constitute a risk for the transmission of antibiotic resistant bacteria. The GCC strategic plan was devised that included five main components, i.e., (a) development of an understanding of the impact of AMR in the GCC member states; (b) put restrictions on the available and limited effective agents with the aim to preserve their efficacy; (c) development of an early identification capacity for emerging multiple drug resistant microbes; (d) limiting the spread of resistant microorganisms by animals, patients, and agriculture means; and (e) encouragement of collaborative research regarding antimicrobial resistance in animals, humans, and the environment [135].

During the Hajj the likelihood of getting a wide diversity of bacteria, especially resistant ones, is significantly high. In the ritual period more than 75% of pilgrims report respiratory tract-related issues. Moreover, since the pilgrims of diverse nationalities and regions can spread microorganisms, especially the resistant ones, through close contact, food, and water, better personal hygiene practices needs to be taught and monitored, in order to reduce the transmission of MDR microbes [136]. The Hajj brings together nearly 183 different nationalities in a mass aggregation of pilgrims. The high temperatures, flu seasons in the northern hemisphere, and other such factors can contribute towards the spread of communicable diseases, especially respiratory tract infections. Viruses like influenza and bacterial infections such as whooping cough and tuberculosis have ample chance of spread. Such spread is not confined to this mass gathering that happens once a year, but the holy cities of Makkah and Madina welcome the Umrah, a short pilgrimage. Thus, the spread of the RTIs is pertinent throughout the year. Compulsory vaccinations include seasonal influenza, diphtheria, poliomyelitis, pertussis, measles, and mumps. The risk factors that account for the spread of tuberculosis include overcrowding, low immune status, old age, undernourishment, and, more importantly, origination from endemic countries. The global rise of MDR-TB issues a serious signal to authorities to take concrete steps to minimize the import and spread of resistant strains [137]. Current reports of emergence of TDR TB (>30 cases) from countries like India, Iran, Italy, and South Africa [80] is a matter of great concern as many pilgrims come from these countries, thus requiring and the development of more specific and stringent SOPs. In a Saudi study, drug resistance was found to be more prevalent in non-Saudi nationals [84]. The spread of drug-resistant organisms is more likely to happen in the months of Hajj and Ramadan, when pilgrim influx is at maximum.

**Figure 4:**
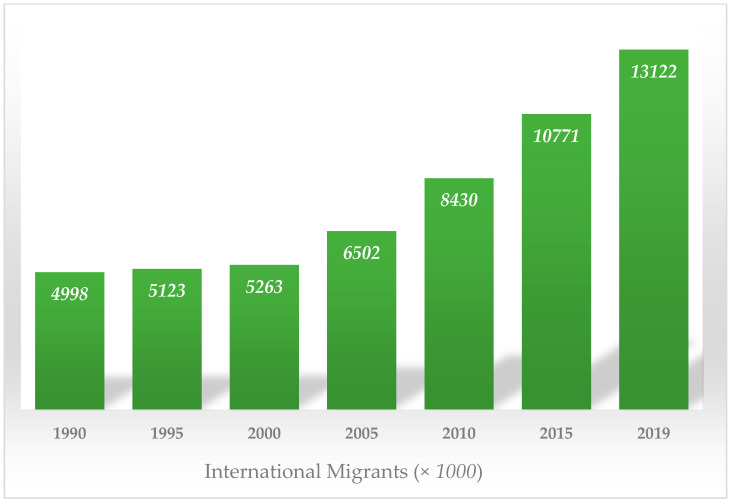
International migrants arriving at KSA from 1990–2019 (Data from United Nations population division’s country profile for international migrants [139]).

With over 50% of pilgrims coming from regions that have 85% of global TB cases (India, Bangladesh, South Asia, Pakistan, and South Africa), the pilgrims of these regions are a particular concern to health authorities of KSA and to the concerned quarters of countries of their origin [138]. The KSA is one of the major countries of the world that welcomes a significant number of migrants, which according to UN international migrant stock 2019 report [139], is between 2.5–5.0 million, 40% or above of the total population of the KSA. The trend in this respect is increasing (Figure 4). A significant number come from underdeveloped countries such as India, Bangladesh, South Asia, and Pakistan. The figures presented in this report are the number of migrants with respect to the countries of origin, indicating India to be the top provider of migrant force followed by Indonesia, Pakistan, and Bangladesh. Thus, pilgrims and migrants who pose a serious concern regarding the TB transmission in the KSA.

It is also important to note that the countries with a high TB burden that share a border with a country with a low TB incidence may change the situation in the adjacent regions of the neighboring countries, which in turn can be transmitted to the rest of the country [140].

Infection from MTB may be acquired as a consequence of travel. In this respect the WHO has given guidelines on tuberculosis and air travel. The risk of spread of tuberculosis in a close indoor vicinity is greater than that outdoors. The risk of spread is greater in longer flights (>8 h) compared to short flights. A detailed travel history needs to be taken with focus on the medical risk factors of patients, especially those with comorbid conditions like renal, pulmonary, and cardiac diseases, advanced age, and low immune status. An early call needs to be relayed to an infectious disease consultant in order to mark travel-related respiratory disease [141].

In this connection, use of simple and inexpensive measures like hand hygiene, use of personal protective equipment (PPE), environmental surfaces cleaning and decontamination, and capitalization of an antimicrobial stewardship program may control infectious disease outbreaks. Similar kind of features can be adopted and implemented in cases of mass gatherings like Hajj and Umrah where the chance of infectious disease outbreaks and transmission is high [142].

The rate of international travel is increasing: although COVID-19-related restrictions caused it to plummet in 2020, with the advent of vaccines it will return. Large gatherings and mass congregations encourage the spread of highly contagious respiratory infections of viral and bacterial origins. The huge rate of pilgrimage to the KSA is a significant public health concern. It can be better apprehended through extensive medical examination prior to and following their visit, mandatory vaccinations, education, and use of PPE, and chemoprophylaxis according to predefined criteria. In this context GCC member states have devised a strategic plan that provides a platform to move forward. It can be further elaborated to better curb infectious diseases that may spread due to mass aggregations such as Hajj and Umrah. Spread of TB also occurs in long-distance flights and living in small vicinities. Nonadherence to ATT is a big challenge for health care personals and to public health authorities. In this regard, the contributing factors are diverse and numerous.

## 8. Public Health Concerns

In a Turkish review, tuberculosis relapse, MDR-TB, family income, patient giving wrong address or shifting without intimation, default history, long duration of treatment, homelessness, stigmatization, going to traditional healers, treatment response, health care staff receptiveness, and poor health education consistently caused non-adherence to TB treatment. The identifiable variables’ influence on the default of TB treatment is for the clinicians who deal with the patients and for health policy makers [143]. Health outcomes are impeded by the stigmatization of TB that affects health care and its delivery. TB-stigma affects social status, inflicts damage on family reputation, and negatively impacts education, employment, and marital prospects. It can also affect mental health and erode patients’ resilience and household wellbeing. The DR-TB has a different relationship with TB stigma in the outcome of disease, being more blamed, shamed, and self-stigmatized due to the HCWs assumption that nonadherence to therapy was its cause [144].

Special segments of society need to be taken care of specifically. For instance, in an Ethiopian study, latent tuberculosis infection (LTBI) prevalence in prisoners was found to be high, which may serve as a source of infection to the general public. Since prisoners live in confinement and, in underdeveloped countries, often in crowded conditions, their handling and follow-up and treatment needs improvement to reduce TB burden and spread [145].

In a large tertiary or quaternary level medical center in San Diego, USA, for foreign-born patients, self-reported nationality and preferred language served as good proxy variable for LTBI screening, prevalence, and treatment rates using electronic health records (EHR) in the identification of missed opportunities for screening and diagnosis of LTBI. EHR can be utilized to end the global TB epidemic [146]. The electronic health information system is a platform that gives support to HCWs in the supply of services in an effective manner and to exchange data among the service providers. Its role is increasing for the surveillance of communicable and non-communicable diseases. It has a role in the surveillance of epidemics and pandemics that can be of significant worth to high disease-burdened countries. This system has the potential to deliver diagnostics to point-of-care and with fewer medical errors [147].

Emergence and spread of resistant TB strains have adversely affected economic, public health, and clinical outcomes. A South African study approximated the cost comparisons of different TB forms, with XDR-TB costing four times more that MDR-TB, and 103 times more than drug-sensitive TB. In Cambodia, MDR-TB cost was four times higher than that of a drug sensitive TB. In a European study, the cost of managing the MDR-TB was 10 times higher than the cost of treating the DS-TB. In an US study, the hospitalization cost per XDR-TB patient was $285,000, 3.5 times higher than the MDR-TB cost that stood at $81,000, while a separate study estimated XDR-TB cost to be $430,000, the MDR-TB to be $130,000, and the DS-TB to be merely $17,000. When these costs were applied to the 364 MDR-TB and nine XDR-TB, huge approximate amounts of $53 million direct costs and $100 million direct plus productivity loss costs came to surface. A prospective dynamic state transition model study from China indicated that by 2050 the MDR-TB incidence and mortality will increase to 60% and 35% respectively, while the same parameters for DS-TB drop by 32% and 41%, respectively, due to the high treatment and cure rates, while the worsening of situation for the MDR-TB is attributable to high transmission, increased prevalence, and in appropriate treatment of LTBI [148]. The price factor in certain countries remains a critical factor for the MDR-TB medicines that are very costly in certain countries. Competitive large-scale manufacturing was suggested [149] that will allow 5–10-times more people to be treated for MDR-TB.

Measures required to fill in the public health gaps with respect to TB include the surveillance of people at high risk of progression to active TB in order to diminish the incidence rate and to improve the control program [150]. Carrying out the GeneXpert^®^ for all the TB cases along with the DST is required to strengthen the TB diagnosis. Other challenges need to be addressed to bring better adherence to TB treatment including better availability of antiTB drugs, improvement in health conditions, disseminating education to patients regarding the disease, and treatment [151].

In a Sudanese study, living in rural areas, poor past treatment, and smoking were found to be associated with the occurrence of MDR-TB [152]. Such variables need to be addressed by public health authorities of the respective regions, since collaborative and coordinated efforts are the only way forward in the control of this disease.

The knowledge of health professionals related to TB is imperative for the patients, for themselves, as well as the global population at large. The knowledge of HCWs serving at Hajj was found average and was associated with the knowledge gaps. Moreover, HCWs were found to lack knowledge of MDR-TB, and also the treatment of TB. The HCWs were found to be less aware of the common symptoms of pulmonary TB [153]. This is important in the context that TB can be an occupational risk factor for HCWs in low- and high-TB incidence countries [154]. The knowledge regarding the significance and use of GeneXpert^®^ MTB/RIF was also poor [153], although this equipment is available in KSA.

In the context of Hajj, the knowledge, attitude, and practices (KAP) regarding TB require improvement in HCWs to avoid any global health issue. Hand hygiene knowledge should be disseminated among HCWs interacting with TB patients. Hands of HCWs may harbor pathogens like methicillin resistant *S. aureus*, MDR-Gram-negative bacteria, *Candida* spp., and vancomycin resistant enterococci [155]; therefore, hand hygiene and use of other PPEs is essential for the HCWs dealing with such patients.

Remotely living TB patients are more likely to use outpatient services instead of inpatient services, thus requiring a tilt in policies towards the underprivileged community living in far-flung areas in order to ensure better utilization of services and to improve therapeutic outcomes [156].

A very useful model in this scenario is taking the US towards TB elimination with declining case numbers and rates. However, expert consultation for complicated and DR-TB were anticipated to increase. The previously established Regional Training and Medical Consultation Centres (RTMCCS) for TB have been transformed to Centres of Excellence, which represent a repository of TB expertise in the US. These centres need to provide consultations on M/XDR-TB to LTBI. Moreover, they need to be made ready for consultations regarding complicated TB cases in order to achieve treatment completion, as well as MDR-TB care at the community level [157]. Establishment of such centres can be a good move towards information dissemination.

Gender, vaccination, socioeconomic status, and tobacco smoking were found to have a significant association with development of pulmonary tuberculosis in the Sudanese population [158]. In another Sudanese study, pharmacy personnel showed good knowledge of TB therapy, the reasons for default, and overall TB cases, but they lacked in knowledge regarding medication safety and had negative attitudes for DOTs [159]. Such kind of bottlenecks need to be eliminated in the way forward to TB elimination.

The TB treatment outcomes improved with use of adherence interventions like material support, psychological support, reminders and tracers, patient education and counselling, and digital health technologies. The trained health workers proved to be more effective in the achievement of DOTs, which has proven significance in the case of HIV [160]. The barriers to adherence to TB treatment and reasons for non-adherence can be multiple. They include health system factors, personal reasons, social and familial factors, and therapy related factors. Health professionals, program implementers, and policy makers should have a sound understanding of such factors to better curb the disease. Close contact with patients, traditional health practitioners, and religious leaders can also play a significant role using their influence on the respective community [161]. All these influencing factors need to be kept in focus to achieve TB elimination.

The community pharmacists, if trained and with sound knowledge of antibiotic dispensing and resistance, can play a vital role in curbing irrational, injudicious, and excessive use. They can work as collaborators between physicians and patients in settling the concerns and educating the patients on the use of antibiotics. They can also be instrumental to physicians by providing them with updates on new guidelines and information on therapeutic categories [162]. This practice can be of great significance with respect to TB treatment that is of long duration, and has associated side effects that need to be communed to patients as they can occasionally result in discontinuation of therapy.

The use of statistical triangulation of data sources yields a strong estimate compared to data from a single source origin. The most important thing from a public health perspective is the capture of all tuberculosis cases through strengthening of surveillance systems at a national level. In this regard, the statistical implications will be revealed in the tracking of TB burden. At the country level, early case detection, accurate diagnostics following rapid initiation of therapy, and meticulous follow-up should be the priorities. Reduction in TB burden will also require efforts to stop smoking, alcohol intake, better management of diabetes, and controlling HIV [163].

Intensive care units are very sensitive yet vulnerable areas of hospitals where the mix of patients infected with easily transmissible diseases can offset infectious organisms, especially the drug resistant ones, to patients (especially the immunocompromised) and to the health care staff. In this context, the facilities need to be adequate and staff need to be trained in cases where patients with resistant organisms like MDR-TB are identified [164].

TB is associated with psychological and social conditions. Psychoeducation has been found to give emotional support to the patients. This effect is improved with such counselling [165]. TB awareness can be improved if information is transmitted through electronic media to women [166]. It can be communicated in an effective manner depending on the education level, wealth, and exposure to electronic media.

Expansion of laboratories and private hospitals have an impact on the TB trends. Although immigration and gross domestic productivity have insignificant relation with TB, financial support is of significant worth in the TB controlling programs especially for illegal immigrants and financially poor [167].

There is a global increase in fluoroquinolone use. Efforts are needed to increase public and health care personnel awareness of antimicrobial drug resistance. For instance, ciprofloxacin and levofloxacin are two very significant FQs that may be subject to inappropriate use in MDR-TB treatment [168]. The treatment of MDR-TB has effects on the physical and mental states of the patients. Therefore, treatment should be done in such manner that the health-related quality of life (HRQoL) of the patient is not affected [169].

The WHO estimates indicate an alarming one-third of world population to have latent tuberculosis. The key risk factors [170] for acquisition of latent TB include assuming TB is a non-serious disease, initial identification in which health authorities were not able to contact, evasion and/or manipulation of immigration examination, self-perception of being at low risk of TB infection with the reason of no contact with tuberculosis infection, issues related to the access the clinic or long wait times, feeling of being stigmatized, having mistrust and unwillingness, lack of awareness campaigns, language and cultural barriers, insufficient knowledge of health-care workers on the need for therapy for tuberculosis, fear of getting deported or immigration status at referral for or at start of treatment, poor coordination among TB program and general health services, presence of other infections such as HIV, syphilis, adverse effects of drugs, and longtime therapy.

The special segments of society, i.e., living in confinement, underprivileged people, immigrants living in substandard conditions, relapse and default history patients, the long duration of treatment, homelessness, going to quacks, M/XDR-TB, stigmatization, health care staff response, and poor health education contribute towards non-adherence to TB treatment resulting in its failure. The problem gets further aggravated in cases of DR-TB that affects the patient psychology, his/her resilience, financials, and other social prospects. In this respect, the EHRs served as a successful platform to facilitate HCWs in controlling the communicable diseases by delivering diagnostics and therapeutics to point-of-care with minimal medical errors. There is a surge in treatment costs, with XDR-TB costing multiple-times more than the MDR-TB, which in turn costs many-times more than DS-TB. Moreover, the independent variables that may influence the development of MDR-TB need to be kept in focus so as to better control the disease progression. The HCWs’ knowledge regarding DR-TB and its treatment is important. It is more crucial in cases of HCWs working near mass gatherings. Access to TB treatment centers is very important, since patients living in remote areas are likely to face failure of desired therapeutic outcomes. Expert opinion and information dissemination are vital as far as the TB and DR-TB are concerned. The US model of RTMCCS is useful in this context as it can be adopted in the regions of high TB prevalence. Since treatment outcomes improve with material and psychological support, reminders and tracers, patient education, and counselling, they need to be utilized subject to their feasibility. Moreover, the influence of religious leaders, traditional healers, close contact with patients, and utilization of iconic personalities for delivering message related to TB are all ploys that may support the TB eradication cause. At the community level the services of community pharmacists can be utilized in an effective manner to collaborate with physicians and patients to better achieve the therapeutic outcomes in TB. Expansion in facilities to better the diagnostics, ideally taking them to the genomic level, improvement of provision of effective therapeutic agents, financial and psychological support to the patients, and all similar actions need to be taken to improve the HRQoL, which is the prime way forward to better cope with the TB.

## 9. Conclusions

TB disease has global prevalence, incidence, and mortality. The mortality predictors include older age, low body weight, delayed start of ATT, rural living, retreatment patients, EPTB infection, low immune status, comorbidities, and coinfections, especially HIV. Moreover, childhood TB is a very concerning issue as well as DM, which presents another huge challenge as it increases the mortality in TB patients thus necessitating its mandatory screening. Moreover, females of childbearing age have been found to be at more risk compared to non-childbearing-age females. DM patients often present with other comorbidities such as heart and renal diseases, thus making the treatment more complex with unfavorable outcomes. When seen in the context of M/XDR-TB the therapeutics become less likely to achieve the desired outcomes.

Eradication of TB by 2030 is part of SDCs set by WHO. The top six TB-burdened countries contribute 60% of new cases to global numbers, therefore requiring stringent governmental policies and public health reforms. The huge global migration from highly-TB-burdened countries affect TB dynamics, especially M/XDR-TB. There is a need to define and monitor TB, especially M/XDR-TB niches. The migratory phenomenon is a problem for economic reasons, and also other factors like poverty, famine, political unrest, and wars which contribute significantly into the spread of highly communicable diseases that, along with comorbid conditions, are a huge public health challenge for the nations that host such refugees and to entire global health systems. There is also risk for countries that have eradicated or are at the verge of eradication of an infectious communicable disease such as TB, thus presenting another public health challenge. The temporary migration in the form of large congregations, and religious and social gatherings contributes to the problem. The Saudi Ministry of Health addressed initiatives to keep the community free from TB, as part of the National Transformation Program (NTP) 2020 and the Saudi Vision 2030.

The use of genetic tools, especially WGS, has proven useful even in unsuspected outbreaks. They are of value in the identification of resistant strains and help in the identification of disease hotspots, which are of important from a public health perspective. The cost of molecular tools is the main bottle neck that needs global assistance from bodies like WHO at an international level while from concerned governments at their respective national levels.

Resistance against the antitubercular drugs is established, and M/XDR-TB is a prime public health concern. Rising resistance against newly-approved drugs is of particular concern to the health and public health authorities. The judicious use of ATT in accordance with well-defined SOPs alongside strict monitoring is the only way to tackle this serious issue. The drug resistance patterns are complex, thus necessitating detailed drug testing prior to therapy. Reliable standard detection and sensitivity assays are pivotal in the success of ATT therapeutics. The most significant current public health concern is the reports of TDR-TB from certain regions of the world. It needs extensive surveillance. Moreover, similar vigilance is required for patients with HIV comorbidity with TB. The biggest concern is the risk of frontline health care professionals’ exposure to resistant mycobacteria, which needs additional tiers in healthcare policy making and implementation.

Current molecular epidemiological data from the KSA suggests a mixed phylogenetic distribution in MDR-TB, indicative of an influx of organisms across the globe due to trade and other migratory activities. Adherence to established guidelines related to TB is a key feature of KSA health policy but the current shift in TB and its resistance dynamics require further strict actions in policy framing and in public health quarters. The mobile outreach team model application at certain areas has yielded remarkable results thus making it a good example to follow for better tackling the situation. However, implementation is needed across the KSA and specifically in major pilgrim- and migrant influx cities like Makkah, Al Madina, and Jeddah. Since the likelihood of the KSA turning into a global TB hotspot is very high, such steps are of prime importance. They are already implemented but need constant revision in light of shifting global TB dynamics. It is pertinent to mention that MTB and other mycobacteria also have zoonotic potential and this aspect needs to be highlighted to public health concerned quarters as resistance phenomena may also spread through a zoonotic course.

Timely initiation of ATT is important in the effective treatment of TB. Correct diagnosis sets the course in planning the right treatment. Drug related considerations like pharmacokinetic surveillance need to be kept in consideration to make optimum use of therapeutics. The use of justifiable empirical therapy can also be done in special circumstances requiring immediate intervention. Treatment options must be logically and rationally utilized along with judicious use of all the complementary and alternative medicines to better achieve treatment outcomes.

The rate of international travel is increasing; although COVID-19-related restrictions caused it to plummet in 2020, with the advent of vaccines it will return. Large gatherings and mass congregations encourage the spread of highly contagious respiratory infections of viral and bacterial origins. The huge rate of pilgrimage to the KSA is a significant public health concern. It can be better apprehended through extensive medical examination prior to and following their visit, mandatory vaccinations, educating and use of PPE, chemoprophylaxis according to predefined criteria. Spread of TB also occurs in long-distance flights and living in small vicinities. Nonadherence to ATT is a big challenge for health care personals and to public health authorities. In this regard, the contributing factors are diverse and numerous.

Special segments of society, i.e., living in confinement, underprivileged people, immigrants living in substandard conditions, relapse and default history patients, the long duration of treatment, homelessness, going to quacks, M/XDR-TB, stigmatization, health care staff response, and poor health education contribute towards non-adherence to TB treatment resulting in its failure. The problem gets further aggravated in cases of DR-TB that affects the patient psychology, his/her resilience, financials, and other social prospects. There is a surge in treatment costs with XDR-TB costing multiple-times more than the MDR-TB, which in turn costs many-times more than the DS-TB. Moreover, the independent variables that may influence the development of MDR-TB need to be kept in focus to better control the disease progression. HCWs’ knowledge regarding DR-TB and its treatment is important. It is crucial in case of the HCWs working close to mass gatherings. Access to TB treatment centers is important, since patients living in remote areas are likely to face failure of desired therapeutic outcomes. Expert opinion and information dissemination are vital as far as TB and DR-TB are concerned. Since treatment outcomes improve with material and psychological support, reminders and tracers, patient education, and counselling, they need to be utilized subject to their feasibility. Moreover, the influence of religious leaders, traditional healers, close contact with patients, and utilization of iconic personalities for delivering messages related to TB are all ploys that may support TB eradication. Expansion in facilities to improve diagnostics, ideally taking them to the genomic level, improvement of provision of effective therapeutic agents, financial and psychological support to the patients, and all similar actions need to be taken in order to improve the HRQoL, which is the best way to cope with the TB. More specific recommendations in this context are:

➢ Pilgrims and migrants both need to be extensively tested for MTB prior to or upon arrival to the KSA.
➢ Comprehensive record of pilgrims’ (for Hajj as well as Umrah) and migrants’ country of origin (CoO) need to be maintained and correlated with the current TB trends in their respective CoO. Preferably digital records need to be maintained for all pilgrims, migrants, and residents who visit.
➢ It is essential to carry out extensive genomic studies with respect to MTB to ascertain the lineages and their susceptibility evaluations. Preferably it needs to be applied to all arrivals, but if this is not possible then can be done on a random basis.
➢ For pilgrims and migrants with a history of TB or belonging to regions with high prevalence of TB, especially with resistance issues, a genetic-based test must be mandated prior to their arrival.
➢ Health authorities of the KSA need to be vigilant of TB trends for the disease trends at home and must regularly coordinate with the concerned quarters of the countries with great influxes of pilgrims and migrants. This data needs to be deciphered in the quota allocation of pilgrims and for the migrant force from a particular country.
➢ Health authorities need to take concrete steps to make available advanced diagnostics capable of extensive drug susceptibility and genetic evaluations, at least to a secondary health care level and ideally at a primary level in order to have better surveillance of TB for the entire country.
➢ The use of PPE by pilgrims, especially masks, should be made essential at all points of mass aggregations.
➢ Pilgrims need to be educated at the use of PPE and made aware of their significance at their CoO prior to their arrival at their destination regions.
➢ Following the success of mobile health care units, the idea needs to be implemented across the country with particular focus on areas of high TB prevalence. Furthermore, the units need to transmit their data and samples to reference labs.
➢ Reference labs having genomic level evaluation facilities needs to be established at provincial levels. All such facilities need to be well coordinated among themselves to generate valid countrywide genomics level with resistance patterns. This may be achieved by the establishment of at least three national tertiary level health and research centres; where whole monitoring, data evaluation, and recommended policy decisions can be made for onward transmission to authorities for approval and implementation.

## Figures and Tables

**Table 1:** Different forms of extrapulmonary tuberculosis (Adapted from Gambhir et al. 2017 [35]).

TB Form	Occurrence
**Tuberculous lymphadenopathy**	Commonly observed form of EPTB in the endemic populations and especially the immunocompromised ones. Commonly affected lymph nodes include cervical, mediastinal, and axillary.
**Abdominal tuberculosis**	It may occur directly (as primary pulmonary TB) or via spread from the primary involving organs like peritoneum, ileocecal junction, colon, liver, spleen, and adrenal glands.
**Peritoneal tuberculosis**	Affects one third of patients and it can be grouped in wet, fibrotic, and dry types
**GI tract tuberculosis**	The abundance of lymphoid tissue; the ileocecal junction is a hotspot for TB.
**Hepatosplenic tuberculosis**	It may present as macronodular that is less common and miliary involvement happening through hepatic artery or portal vein in case of GI lesions.
**Adrenal tuberculosis**	Most common endocrine glands involved in TB that leads to primary adrenal insufficiency in case of adrenal cortex involvement, which if exceeds >90% may result in life endangering Addisonian crisis
**Renal tuberculosis**	Nearly 15–20% EPTB cases account for renal TB
**Male genital organs TB**	The seminal vesicles or prostate gland are generally involved, with rare involvement of the epididymis and testes
**Female genital organs TB**	Nearly 1.5% of females’ genitals are affected by the TB through lymphatic or hematogenous route.
**Musculoskeletal TB**	Hematogenous spread from lungs or from activation of dormant infection in joints or bones following a trauma
**Tubercular spondylitis**	Vertebrae are involved, with most common involvement of the lower thoracic and upper lumbar vertebrae
**Tubercular arthritis**	Majority (90%) cases have large joints like knee and hip involved, with less common involvement of joints of shoulders, elbow, and sacroiliac region
**Tubercular osteomyelitis**	Large bones of extremities, such as the tibia and femur, are commonly involved but the small bones of the hands and feet may also be affected
**Tubercular dactylitis**	It is more common in children with painless involvement of short tubular bones of hands and feet
**Central nervous system TB**	The hematogenous or direct extension from a localized infection CNS involvement is common in highly endemic regions
**Tubercular leptomeningitis**	Presence of thick tubercular exudate in the base of brain in the subarachnoid region is a distinguishing feature. It may hamper the flow of CSF. With ischemic infarcts developing due to arterial involvement and cranial nerves getting affected too.
**Tubercular pachymeningitis**	This is a rather rare occurrence with plaque-like regions of pachymeningeal enhancement seen in CT scans
**Tuberculoma**	Presentation is through solitary, multiple or lesions of frontal and parietal lobes
**Tubercular abscess**	A large solitary lesion that may be multiloculated, vasogenic edema and pus-filled centers, and vascular granulated tissues
**Rhomboencephalitis**	In this condition of the hind brain gets affected with tuberculoma
**Tubercular encephalopathy**	Commonly seen in children and infants that may be due to DTH reactions to a tubercular protein causing extensive damage to white matter
**Spinal and meningeal TB**	Linear enhancing exudates along the spinal cord region
**Tubercular ostomastoiditis**	Painless or with pain chronic otorrhea with an intact tympanic membrane, purulent discharge, and ossicular erosion are the common presenting features
**Tubercular mastitis**	Described by Sir Astley Cooper as “Scrofulous swelling of the bosom” [36]. It is a rare occurrence but is having increasing incidence that is presented either as nodular or multifocal disease
**Cardiac TB**	A rare infection that involves predominantly the pericardial and myocardial muscles, which may extend to endocardium
**TB in immunocompromised states**	The incidence of TB in immunocompromised states especially in HIV infections is very high that is very critical in the M/XDR perspectives

(EPTB = Extrapulmonary tuberculosis; GI = Gastrointestinal; TB = Tuberculosis; CNS = Central nervous system; CSF = Cerebrospinal fluid; CT = Computed tomography; DTH = Delayed type hypersensitivity; HIV = Human immunodeficiency virus; M/XDR = Muti/Extremely drug resistance).

**Table 2:** Different Tests and Techniques used in the Phenotypic, Genotypic detection, and Susceptibility evaluation of *Mycobacterium tuberculosis*.

Diagnostic Technique Type	Diagnostic Techniques with Advantages	Shortcomings/Limitations
***Rapid Tests***	*Sputum microscopy* ✓ WHO recommended gold-standard ✓ Inexpensive	Requires a reasonably high number 5 × 10^3^–10^4^ of bacilli per mL for detection Discrimination between different Mycobacteria species not possible
	*Fluorescent microscopy* ✓ WHO recommended	Low sensitivity resulting in false negatives Discrimination between different Mycobacteria species not possible
	*Histopathological test* ✓ Used for Extra Pulmonary TB cases identification	Requirement of trained personnel for sampling as well as the facilities are the major bottleneck
***Standard Diagnostic Tests***	*Culturing on Solid Media* ✓ Highly sensitive as it requires merely 10^1^–10^2^ bacilli/mL ✓ Phenotypic characterization and Drug Susceptibility Tests (DST) can be undertaken ✓ Supply adequate organisms that are required for molecular analyses ✓ WHO recommended	Higher Turnaround Time (TT) as it takes 6–8 weeks to five results thereby diagnoses as well as treatment initiation get delayed making the clinical picture further grim Biosafety level 3 (BSL-3) facilities with trained personnel are required Cultures contamination may happen and require an effective decontamination step that also affects the target microbe.
	*Culturing on Liquid Media* ✓ Growth is rapid in liquid media, i.e., TT ≈ 10 days ✓ Semiautomated as well as automated systems are available ✓ High sensitivity that may effectively diagnose smear negative samples ✓ WHO recommended ✓ A WHO accepted standard to evaluate the susceptibility of 1st and 2nd line antitubercular drugs	Low sensitivity in case of EPTB and childhood TB as a consequence of paucibacillary nature of the assay Cultures are highly prone to other microbial growths and require effective decontamination step that effects the target microbe also. Biosafety level 3 (BSL-3) facilities with trained personnel are required
***PCR based Diagnostic Tests***	*Line Probe Assay (LPA)* ✓ Highly sensitive and specific ✓ Used for species level detection as well as evaluation of resistance against 1st line and 2nd line antitubercular drugs	Lack of mixed cultures distinction is the major shortcoming Smear negative specimens cannot be evaluated through LPA
	*Cartridge based Nucleic Acid Amplification Tests (NAATs)* ✓ High sensitivity and specificity with short TT ✓ Simultaneous testing for TB as well as RIF resistance	Expensive and needs subsidization from health authorities
	*XPert^®^ MTB/RIF assay* ✓ WHO recommended rapid test with around 2 h reporting time ✓ Minimal infrastructure requirement ✓ Good sensitivity and specificity	Expensive and needs subsidization from health authorities
	*Loop-mediated Isothermal Amplification* ✓ Single step DNA amplification and gene detection method ✓ Rapid with TT of ≈40 min	Less specific Drug resistance evaluation cannot be done
	*Gene Drive* ✓ Simultaneous determination of TB and RIF resistance	Less accurate
	*Whole genome sequencing (WGS)* ✓ Complete genome is sequenced ✓ Rare mutations and heteroresistance is defined ✓ Prespecified targets are not required	Expensive technique Skilled personnel required for experimentation as well as interpretation
	*Spoligotyping* ✓ Quick detection and typing of MTB ✓ Useful for surveillance of TB transmission and to control spread ✓ Presence or absence of 35–43 spacer oligonucleotides	Compared to WGS less expensive but skilled personnel are required for execution and interpretation
***Future Advanced Diagnostic Tests undergoing Experimentations***	Constant research is underway to look for effective, cost-efficient, reproducible, highly sensitive, safe and requiring very less infrastructure tools. Some of the projects that are at different stages of experimentations are: ✓ Automated microscopy platforms ✓ GeneXpert Ultra ✓ Volatile Organic Acids (VOC) assays ✓ Lipoarabinomannan (LAM) assay ✓ Nanoparticles based assays ✓ Lab-on-a-chip platform ✓ TRCReady 80 ✓ Upgraded Tuberculin Skin Test ✓ Interferon-γ (INF-γ) release assay ✓ Next Generation Sequencing (NGS) platforms	
